# Characterisation of Non-Carbapenemase-Producing Carbapenem-Resistant *Klebsiella pneumoniae* Based on Their Clinical and Molecular Profile in Malaysia

**DOI:** 10.3390/antibiotics11111670

**Published:** 2022-11-21

**Authors:** Yee Qing Lee, Sasheela Sri La Sri Ponnampalavanar, Chun Wie Chong, Rina Karunakaran, Kumutha Malar Vellasamy, Kartini Abdul Jabar, Zhi Xian Kong, Min Yi Lau, Cindy Shuan Ju Teh

**Affiliations:** 1 Department of Medical Microbiology, Faculty of Medicine, University of Malaya, Kuala Lumpur 50603, Malaysia; 2 Department of Medicine, Faculty of Medicine, University of Malaya, Kuala Lumpur 50603, Malaysia; 3 School of Pharmacy, Monash University Malaysia, Jalan Lagoon Selatan, Bandar Sunway 47500, Malaysia

**Keywords:** all-cause in-hospital mortality, Charlson Comorbidity Index (CCI), efflux pump, non-carbapenemase-producing carbapenem-resistant *Klebsiella pneumoniae*, porin loss, resistance genes

## Abstract

Non-carbapenemase-producing carbapenem-resistant *Klebsiella pneumoniae* (NC-CRKP) confers carbapenem resistance through a combination of chromosomal mutations and acquired non-carbapenemase resistance mechanisms. In this study, we aimed to evaluate the clinical and molecular profiles of NC-CRKP isolated from patients in a tertiary teaching hospital in Malaysia from January 2013 to October 2019. During the study period, 54 NC-CRKP-infected/colonised patients’ isolates were obtained. Clinical parameters were assessed in 52 patients. The all-cause in-hospital mortality rate among NC-CRKP patients was 46.2% (24/52). Twenty-three (44.2%) patients were infected, while others were colonised. Based on the Charlson Comorbidity Index (CCI) score, 92.3% (48/52) of the infected/colonised patients had a score of ≥ 1. Resistance genes found among the 54 NC-CRKP isolates were *bla*_TEM_, *bla*_SHV_, *bla*_CTX-M_, *bla*_OXA_, and *bla*_DHA_. Porin loss was detected in 25/54 (46.3%) strains. None of the isolated strains conferred carbapenem resistance through the efflux pumps system. In conclusion, only 25/54 (46.3%) NC-CRKP conferred carbapenem resistance through a combination of porin loss and the acquisition of non-carbapenemase resistance mechanisms. The carbapenem resistance mechanisms for the remaining strains (53.7%) should be further investigated as rapid identification and distinction of the NC-CRKP mechanisms enable optimal treatment and infection control efforts.

## 1. Introduction

Carbapenem-resistant Enterobacterales (CRE) have been listed as an urgent threat [1]. CREs are resistant to at least one of the carbapenem antibiotics (ertapenem, meropenem, imipenem, or doripenem) or produce a carbapenemase, which is an enzyme that can hydrolyse β-lactam and allows bacteria to be resistant to carbapenem antibiotics [2]. Carbapenem-resistant *Klebsiella pneumoniae* (CRKP) is the predominant pathogen among epidemic and endemic CRE infections that carries a high mortality rate [3,4]. CRKP includes both carbapenemase-producing (C-CRKP) and non-carbapenemase-producing (NC-CRKP) strains. C-CRKP confers carbapenem resistance through carbapenemase production [5]. NC-CRKP confers carbapenem resistance through a combination of chromosomal mutations (e.g., porin gene mutation, overproduction of efflux pump, and/or alteration in penicillin-binding protein) and acquired non-carbapenemase resistance mechanisms (acquisition or upregulation of a β-lactamase such as extended-spectrum β-lactamase (ESBL) or AmpC β-lactamase) [2,6].

Based on our previous study and unpublished data from the Infection Control Department (ICD) of the University of Malaya Medical Centre (UMMC), most of the CRKP isolated in our hospital setting were C-CRKP [7]. Changing trends of genotypic characteristics among C-CRKP leading to different phenotypic traits have been observed. In 2013, the predominant carbapenemase gene of C-CRKP was *bla*_OXA-48_ (70.6%), followed by *bla*_KPC-2_ (29.4%), *bla*_NMC-A_ (11.8%)_,_ *bla*_IMP-8_ (5.9%), and *bla*_NDM-1_ (5.9%) genes [8]. In 2014, only two carbapenemase genes were detected. The *bla*_NDM_ (56.0%) was the predominant gene, followed by *bla*_OXA-48_ (36.0%) [9]. In 2015, the predominant carbapenemase gene of C-CRKP had reverted to *bla*_OXA-48_ (79.1%), followed by *bla*_NDM_ (40.9%) and *bla*_OXA-232_ (0.9%) [9]. In 2016, the predominant carbapenemase gene was still *bla*_OXA-48_ (75.6%), followed by *bla*_NDM_ (22.2%) [10]. Both *bla*_OXA-48_ (43.8%) and *bla*_NDM_ (43.8%) were the predominant carbapenemase genes of C-CRKP detected in 2017 [10]. However, there is a lack of epidemiology data on NC-CRKP or their mechanisms of resistance in our hospital.

A retrospective study conducted in South Texas between 2011 and 2019 reported that the majority of CRE (59.0%, 58/99) isolates were non-carbapenemase-producing strains [11]. A cohort study conducted in Maryland from 2016 to 2021 also revealed that 54.0% (327/603) of CRE isolates were non-carbapenemase-producing strains, with *Klebsiella pneumoniae* as the predominant pathogen [12]. A study conducted in Taiwan from January 2013 to December 2018 revealed that 86.9% (86/99 isolates) of the CRE bacteremia specimens were non-carbapenemase-producing strains [13]. Additionally, a study conducted by six Singapore public sector hospitals between December 2013 and April 2015 reported that 35.3% (88/249 subjects) of their recruited subjects had non-carbapenemase-producing CRE [14]. Nonetheless, little is known about the pathogenicity, persistence, and clinical outcomes of the NC-CRKP in Malaysia.

A review study conducted in the United States reported that NC-CRKP were less transmissible than C-CRKP and can be easily eliminated in vivo by the immune system [15]. A cohort study of 83 patients with monomicrobial CRE bacteremia conducted in the United States in 2013-2016 reported that carbapenemase-producing CRE was more virulent with a higher mortality rate (32.4%) than non-carbapenemase-producing CRE (13.0%) [16]. Nonetheless, the burden of NC-CRKP should not be underestimated as a study carried out between 2008-2011 in Italy reported that the mortality rate of patients with NC-CRKP (37.9%) was similar to that of KPC-producing (38.9%) CRKP [17]. A multicentre study in Taiwan also reported a high 14-day mortality rate (27.3%) among 99 NC-CRKP patients [18]. In this study, we sought to determine the prevalence, genotypic characteristics, and phenotypic profile of NC-CRKP as well as the risk factors of NC-CRKP associated with all-cause in-hospital mortality in a tertiary teaching hospital in Malaysia.

## 2. Results

### 2.1. Bacterial Strains Collection

**Figure 1:**
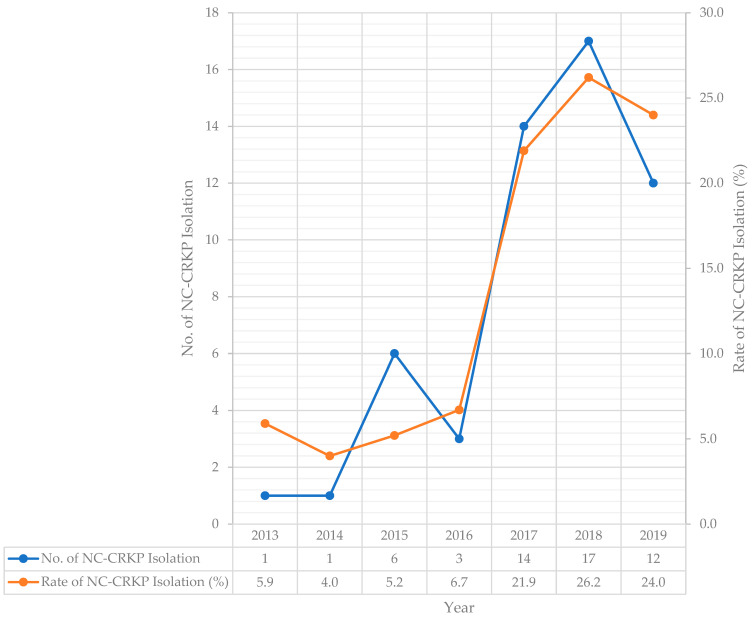
Isolation of NC-CRKP (clinical and screening) among hospitalised patients in UMMC, Malaysia from 2013 to October 2019.

From 2013 to 2019, a total of 381 CRKP were collected from patients’ clinical and screening samples. An increase in NC-CRKP strains has been observed over the seven years, as shown in Figure 1. In this study, 54 NC-CRKP were isolated from UMMC patients from January 2013 to October 2019. In 2013 and 2014, 5.9% and 4.0% of the CRKP isolated from UMMC patients were NC-CRKP. An increase in the rate of NC-CRKP strains was observed from 5.2% in 2015 to 6.7% in 2016, even though the reported CRKP cases had decreased from 115 cases in 2015 to 45 cases in 2016. The rate of NC-CRKP strains had increased to 21.9% in 2017 and 26.2% in 2018, but a slight decline to 24.0% in 2019.

The rate of NC-CRKP isolation was calculated as a percentage (%) of the total number of NC-CRKP strains isolated per year over the total number of CRKP strains.

### 2.2. Determination of MIC Profiles

Based on the broth microdilution method (Table 1), all strains were resistant to ertapenem and ciprofloxacin. Broth microdilution also revealed that 61.1% and 33.3% of the strains were susceptible to imipenem and meropenem, respectively. The highest MIC value of ertapenem, imipenem, meropenem, and ciprofloxacin was >256 µg/mL, 128 µg/mL, 256 µg/mL, and >512 µg/mL, respectively. Only two strains were colistin-resistant with a MIC of 32 µg/mL, while the remaining were intermediate to colistin (The CLSI guidelines do not give any interpretive MIC breakpoint for susceptible). For carbapenem resistance among all 54 NC-CRKP, 16 strains (29.6%) were mono-resistant to ertapenem (Table 2).

### 2.3. Antimicrobial Susceptibility Data

All 54 strains were resistant to amoxicillin/clavulanate, ampicillin, and cefuroxime (Table 3). All strains were resistant to at least three antimicrobial classes and were therefore classified as multidrug-resistant (MDR) strains [19].

### 2.4. Determination of Resistance Genes and Porin-Associated Genes

None of the 54 NC-CRKP harboured the five major carbapenemase genes targeted for detection in this study, which were *bla*_NDM_, *bla*_OXA-48_, *bla*_IMP_, *bla*_VIM_, and *bla*_KPC_ (Table 4). Each strain harboured at least one non-carbapenemase β-lactamase gene targeted for detection in this study. Resistance genes found among the 54 NC-CRKP isolates were *bla*_DHA_, *bla*_TEM_, *bla*_SHV_, *bla*_CTX-M,_ and *bla*_OXA_ genes (Table 4). In total, deletion of porin-associated genes was detected in 46.3% (25/54) of the NC-CRKP (Table 2 and Table 4). None of the 54 strains had ompK37 porin loss.

### 2.5. MIC Reduction Assay with Efflux Pump Inhibitor Tests

For the MIC of imipenem, meropenem, and ertapenem after the addition of PAβN, no significant reduction was observed (Table 5). Hence, no efflux contribution of PAβN-inhibited efflux pumps to carbapenem resistance was detected among the 54 strains. However, the PAβN-inhibited efflux pump contributed to the resistance to ciprofloxacin, as a four-fold decrease in the MIC of ciprofloxacin was observed in five strains, after the addition of PAβN (Table 5). In addition, 46.3% (25/54 strains) of the NC-CRKP showed a significant increase (≥ 4-fold) in the MIC of at least one carbapenem in the presence of PAβN (Table 5 and Table A1 in Appendix A). After the addition of PAβN, 44.4% (24/54 strains) of the NC-CRKP showed a two-fold increase in the MIC of at least one carbapenem, while the remaining strains (9.3%, 5/54 strains) showed no change in the MIC of carbapenem.

### 2.6. Pulsed-Field Gel Electrophoresis

**Figure 2:**
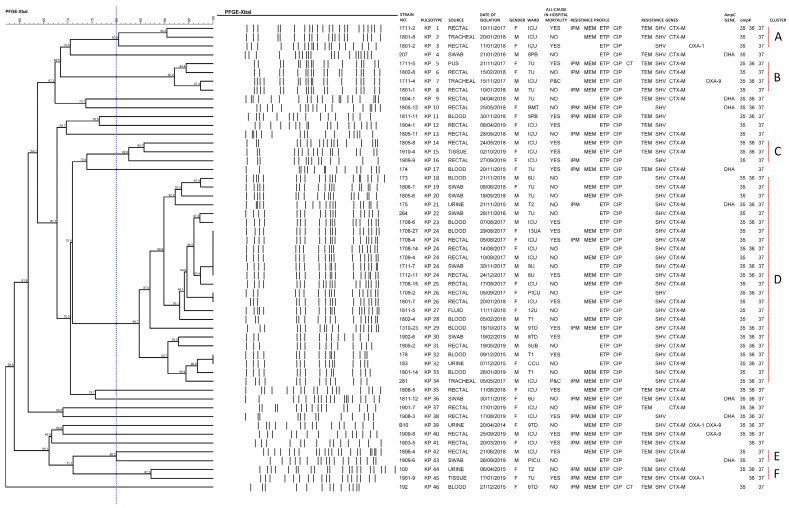
Dendrogram of 53 strains. Gender: Female (F) or male (M). Resistance profile: Imipenem (IPM), meropenem (MEM), ertapenem (ETP), ciprofloxacin (CIP), and/or colistin (CT). Presence of *AmpC* β-lactamase gene: *bla*_DHA._ Presence of other non-carbapenemase β-lactamase genes: *bla*_TEM_, *bla*_SHV_, *bla*_CTX-M_, *bla*_OXA-1_, and/or *bla*_OXA-9._ Presence of porin-associated genes: *ompK35*, *ompK36*, and/or *ompK37*. Pulsotype: Strains that exhibited 100.0% similarity. Cluster: Pulsotypes that had 80.0% similarity. “P&C” is an abbreviation for private and confidential.

For PFGE dendrogram analysis, one untypeable strain (Strain no.: 285) was excluded. Among 53 NC-CRKP, 46 pulsotypes were detected and assigned as KP 1 to KP 46. These 46 pulsotypes were grouped into six clusters (Clusters A, B, C, D, E, and F) based on an 80.0% similarity cut-off (Figure 2). All six clusters harboured the *bla*_SHV_ gene.

Cluster D was the dominant strain cluster (with 24 strains) detected from 2013 to 2019. Cluster D was resistant to ETP, CIP, AMC, AMP, CXM, CTX, CAZ, and CRO. Loss of ompK36 porin was detected among 11 strains in cluster D. Cluster D was comprised of pulsotypes KP 18 to KP 34. KP 24 was the predominant pulsotype found in six patients admitted to UMMC from August to December 2017. Pulsotype KP 24 harboured *bla*_SHV_ and *bla*_CTX-M_ genes. Pulsotype KP 24 was resistant to ETP and CIP. A loss of ompK36 porin was detected among three strains in pulsotype KP 24.

Cluster A composed of pulsotypes KP 1 to KP 3 isolated from 2017 to 2018. Cluster A was resistant to ETP, CIP, AMC, AMP, CXM, CTX, and GM. A loss of ompK36 porin was detected in two strains from cluster A.

Cluster B was characterised by pulsotypes KP 5 to KP 8 isolated from 2017 to 2018. In addition to the *bla*_SHV_ gene, cluster B harboured *bla*_TEM_ and *bla*_CTX-M_ genes. Cluster B was resistant to ETP, CIP, AMC, AMP, CXM, CTX, CAZ, CRO, and MEM, while susceptible to GM. A loss of ompK36 porin was detected in two strains from cluster B.

Cluster C was composed of pulsotypes KP 14 to KP 16 isolated from 2018 to 2019. Cluster C was resistant to ETP, CIP, AMC, AMP, CXM, and CAZ, while susceptible to GM. Only one strain in cluster C had ompK36 porin loss.

Cluster E was characterised by pulsotypes KP 42 isolated in 2018 and KP 43 isolated in 2019. Cluster E was resistant to ETP, CIP, AMC, AMP, CXM, CTX, CAZ, and CRO, while susceptible to GM and IPM. Cluster E encountered ompK36 porin loss.

Cluster F was composed of pulsotypes KP 44 isolated in 2015 and KP 45 isolated in 2019. In addition to the *bla*_SHV_ gene, cluster F harboured *bla*_TEM_ and *bla*_CTX-M_ genes. Cluster F was resistant to ETP, CIP, AMC, AMP, CXM, CTX, CAZ, CRO, IPM, and MEM, while susceptible to GM. Only one strain (pulsotype KP 45) in cluster F had ompK35 porin loss.

Additionally, 15 strains that were isolated in 2014 (pulsotype KP 39), 2015 (pulsotype KP 17 and KP 46), 2016 (pulsotypes KP 4), 2018 (pulsotypes KP 9, KP 10, KP 11, KP 13, KP 35 and KP 36) and 2019 (pulsotypes KP 12, KP 37, KP 38, KP 40 and KP 41) showed distinct pulsotypes. These strains could not be grouped into clusters A to F. All 15 strains were resistant to ETP, CIP, AMC, AMP, CXM, CTX, and CRO. A loss of both ompK35 and ompK36 porins was detected in pulsotype KP 17. Pulsotype KP 41 had ompK35 porin loss. Pulsotypes KP 4, KP 12, KP 13, and KP 46 had ompK36 porin loss.

### 2.7. Clinical Data and Statistical Analysis

Of the 54 NC-CRKP patients, two patients were excluded from the statistical analysis due to the restricted access to highly confidential patients’ demographic and clinical data. All 52 infected/colonised patients had invasive devices in situ before NC-CRKP was isolated. The all-cause in-hospital mortality rate was 46.2% (24/52). 23 (44.2%) patients were infected with NC-CRKP, while others were colonised. Since antimicrobial treatment was only prescribed for infected patients, the independent variables like empiric treatment and definitive therapy were included only in the infection model to analyse the all-cause in-hospital mortality risk among 23 NC-CRKP infected patients.

When considered separately in determining the risk factors of NC-CRKP association with all-cause in-hospital mortality, a total of two parameters, including previous cephems/cephalosporins exposure and previous carbapenem exposure, were found to be significant with *p* < 0.050 (Table 6).

Independent variables with a *p*-value of 0.150 or less in the univariate analysis were selected for the multivariate binary logistic regression model to evaluate the risk for all-cause in-hospital mortality among NC-CRKP-infected/colonised patients (Table 7). The binary logistic regression model achieved an overall correct classification of 82.7% with a χ^2^ value of 32.804 and a *p*-value of less than 0.010 (*p* = 0.003). In the multivariate binary logistic regression analysis, no statistically significant risk factor was found.

## 3. Discussion

This study showed an increasing trend of NC-CRKP prevalence from 1 strain in 2013 to 12 strains in 2019 and attained the highest (17 strains) isolation in 2018. There was a notable rise in NC-CRKP strains over these seven years, especially from 2016 (3 cases) to 2017 (14 cases). Among 52 NC-CRKP-infected/colonised patients, the all-cause in-hospital mortality rate was 46.2%. The mortality rate of NC-CRKP-infected/colonised patients in our study was higher as compared to the United States (13.0%) [16], Italy (37.9%) [17], and Taiwan (27.3%) [18]. For NC-CRKP-infected patients (44.2%, 23/52) and NC-CRKP-colonised patients (55.8%, 29/52), the all-cause in-hospital mortality rate was 52.2% and 41.4% respectively. Based on the CCI score, 92.3% (48/52) of the infected/colonised patients had comorbidity with a score of ≥1. For NC-CRKP-infected/colonised patients with CCI scores of ≥5 (severe; 61.5%, 32/52), the all-cause in-hospital mortality rate was 53.1% (17/32). Compared with severe and moderate CCI patients, patients with lower CCI scores (mild/no comorbidity) had a higher survival rate. Previous evidence suggested that NC-CRKP were confined to individuals and environments with very high levels of antimicrobial selection pressure [20], especially those with heavy use of carbapenem [17]. In the present study, NC-CRKP-infected/colonised patients with previous carbapenem exposure had a higher all-cause in-hospital mortality rate (63.0%, 17/27) than patients with previous cephems/cephalosporins exposure (33.3%, 12/36). Of 27 (51.9%) NC-CRKP-infected/colonised patients with previous carbapenem exposure, 75.0% (6/8) of the infected patients died, while 57.9% (11/19) of the colonised patients died. Among the 36 infected/colonised patients with previous cephems/cephalosporins exposure, 47.1% (8/17) of the infected patients died, whereas 21.1% (4/19) of the colonised patients died. NC-CRKP may be more frequently acquired endogenously through antimicrobial selective pressure as they were resistant to carbapenem via the non-enzymatic carbapenem-resistant mechanisms, while C-CRKP was more frequently acquired exogenously through horizontal gene transfer [15]. The horizontal gene transfer by mobile genetic elements contributes to the persistence of carbapenemase genes among C-CRKP in hospitals despite aggressive infection control [21]. However, antimicrobial selective pressure also predisposes the microorganism to be more susceptible to horizontal gene transfer events, which promotes penetration of the mobilome into new bacterial hosts and leads to the dissemination of antimicrobial resistance [22,23].

Broth microdilution revealed that all 54 strains were resistant to ertapenem, while 61.1% and 33.3% of the strains were susceptible to imipenem and meropenem, respectively. This was similar to another NC-CRKP study in which the loss of susceptibility was more remarkable for ertapenem, followed by meropenem and imipenem [24]. All 54 strains were resistant to ETP, CIP, AMC, AMP, and CXM, and hence fulfiled the criteria as MDR strains. Nineteen strains were resistant to all three carbapenems. Seventeen strains were resistant to meropenem and ertapenem, while two strains were resistant to imipenem and ertapenem. Additionally, 29.6% (16/54) of the strains were mono-resistant to ertapenem. A cohort study in the United States reported that ertapenem-mono-resistant CRE rarely has carbapenemase genes [25]. In addition to being resistant to ETP, CIP, AMC, AMP, and CXM, these 16 ertapenem-mono-resistant strains were also resistant to CTX and harboured the *bla*_SHV_ gene. Only 6 strains out of these 16 strains had ompK36 porin loss, while ompK35 and ompK37 porin loss were not detected in these 16 strains. This suggested that ertapenem and cefotaxime resistance were not due to the loss of ompK35 porin. A study reported that the loss of ompK35 porin alone may not confer high-level resistance to ertapenem and may not affect susceptibility to imipenem and meropenem [24]. Additionally, a previous study also reported that a decrease in expression of ompK36 porin, but not ompK35 porin, was statistically associated with individual carbapenem resistance as the former facilitates the penetration of cefotaxime, cefoxitin, and carbapenem [26]. It is noteworthy that ertapenem resistance normally arises from a combination of non-carbapenemase β-lactamase with altered porins and can be controlled by non-ertapenem carbapenem [27,28]. The majority (68.8%, 11/16) of these ertapenem-mono-resistant strains belonged to the dominant cluster D in PFGE.

Among the 54 NC-CRKP in this study, both *bla*_SHV_ and *bla*_CTX-M_ were the predominant genes between 2013–2016, but *bla*_SHV_ was the predominant gene between 2017–2019. Overall, the most prevalent non-carbapenemase β-lactamase gene among the 54 NC-CRKP in this study was *bla*_SHV_ (53/54, 98.1%), followed by *bla*_CTX-M_ (46/54, 85.2%)_,_ *bla*_TEM_ (24/54, 44.4%), *bla*_DHA_ (8/54, 14.8%), *bla*_OXA-1_ (3/54, 5.6%), and *bla*_OXA-9_ (3/54, 5.6%) genes. Although *bla*_SHV_ is ubiquitous in *K. pneumoniae*, previous studies reported the absence of this enzyme [29,30]. While *bla*_CTX-M_ is an ESBL gene, not all *bla*_TEM_ and *bla*_SHV_ genes are necessarily ESBL genes [31]. Hence, further sequencing is needed to confirm whether they are narrow- or extended-spectrum β-lactamase genes. On the other hand, the *bla*_OXA-1_ and *bla*_OXA-9_ genes are narrow-spectrum class D β-lactamase genes [32]. Among all 54 MDR strains, 85.2% (46/54) of the strains carried the *bla*_CTX-M_ ESBL gene (5 strains co-harboured *AmpC* β-lactamase gene), while 5.6% (3/54) of the strains carried *AmpC* β-lactamase gene. The remaining five strains (9.3%) carried other non-carbapenemase β-lactamase genes. However, the spectrum could not be confirmed as the sequencing of *bla*_TEM_ and *bla*_SHV_ genes were not performed. The *bla*_DHA_ was the only *AmpC* β-lactamase gene detected in 14.8% (8/54) of the NC-CRKP. The *bla*_DHA_ is generally regarded as plasmid-encoded due to the absence of the chromosomal *bla*_AmpC_ gene in the genome of *K. pneumoniae* [33]. Plasmid-mediated *AmpC* β-lactamase genes such as *bla*_DHA-1_ are inducible by β-lactam [34] and can be expressed in high levels constitutively while downregulating inflammation to depress the immune response [35]. Additionally, previous studies reported that the majority of NC-CRKP had porin deficiency [18,36] which may cause nutrient uptake impairment and lower metabolic fitness [37]. Nevertheless, only 46.3% (25/54) of the NC-CRKP in this study had porin loss. The loss of ompK35 or ompK36 porins was detected in 5.6% and 42.6% of the NC-CRKP respectively. None of the 54 strains had ompK37 porin loss. Among the 25 strains that exhibited porin loss, 84.0% (21/25) of the strains carried the *bla*_CTX-M_ ESBL gene (2 strains co-harboured *AmpC* β-lactamase gene), while 4.0% (1/25) of the strains carried *AmpC* β-lactamase gene. The remaining three strains (12.0%) carried other non-carbapenemase β-lactamase genes targeted for detection in this study. They were either narrow- or extended-spectrum β-lactamase genes as the sequencing of *bla*_TEM_ and *bla*_SHV_ genes were not conducted in this study to confirm their spectrum.

In addition to ETP, CIP, AMC, AMP, and CXM, all *bla*_TEM_-producing strains were resistant to CTX, while all *bla*_CTX-M_-producing strains were resistant to CAZ and CTX. All strains that harboured the *bla*_DHA_ gene co-carried the *bla*_SHV_ gene and were resistant to ETP, CIP, AMC, AMP, CXM, CAZ, CTX, and CRO. All strains that harboured the *bla*_OXA-9_ gene also carried the *bla*_SHV_ and *bla*_CTX-M_ genes. Hence, all *bla*_OXA-9_-producing strains were resistant to ETP, CIP, AMC, AMP, CXM, CAZ, CTX, CRO, and MEM. None of the *bla*_OXA-9_-harbouring strains had porin loss. All strains that harboured the *bla*_OXA-1_ gene co-carried *bla*_SHV_ gene and were resistant to ETP, CIP, AMC, AMP, CXM, and CTX. Strains exhibiting ompK35 porin loss (5.6%, 3/54 strains) also harboured *bla*_TEM_, *bla*_SHV,_ and *bla*_CTX-M_ genes. They were resistant to all three carbapenems (ETP, IPM, MEM), CIP, AMC, AMP, CXM, CAZ, CTX, and CRO, but susceptible to GM. A loss of ompK35 porin usually contributes to the emergence of antimicrobial resistance in ESBL-producing strains and may favour the selection of additional mechanisms of resistance, including the loss of ompK36 and/or active efflux [38]. In addition to ETP, CIP, AMC, AMP, and CXM, all imipenem-resistant strains were resistant to CAZ, while all meropenem-resistant strains were resistant to CAZ and CTX. All gentamicin-resistant strains were resistant to ETP, CIP, AMC, AMP, CXM, and CTX. All imipenem-resistant strains and gentamicin-resistant strains harboured the *bla*_SHV_ gene.

In this study, the PAβN-inhibited efflux pump contributed to the resistance to ciprofloxacin, as a four-fold decrease in the MIC of ciprofloxacin was observed in five strains, after the addition of PAβN. PAβN is one of the most extensively studied compounds which acts as a peptidomimetic efflux pump inhibitor that combines with fluoroquinolone antibiotics to overcome efflux-mediated multidrug resistance [39]. However, PAβN did not reduce carbapenem resistance among all these 54 NC-CRKP. Therefore, the efflux system was unlikely to be involved in carbapenem resistance, which was in accordance with previous studies [40,41,42]. In addition, 46.3% (25/54 strains) of the NC-CRKP showed a significant increase (≥4-fold) in the MIC of at least one carbapenem in the presence of PAβN. According to Saw et al. (2016), the effect of PAβN on the MIC of carbapenem was caused by the altered expression of outer membrane porins, as their clinical isolates (15.1%, 13/86 strains) with increased ertapenem resistance in the presence of PAβN had altered porin expression, whereas those (3.5%, 3/86 strains) with no differences in ertapenem MIC value after the addition of PAβN did not. Careful evaluation of new efflux inhibitors is necessary to ensure that antimicrobial-resistant bacteria do not develop increased resistance to clinically important antimicrobials [42].

Since all the strains isolated had at least one non-carbapenemase β-lactamase gene targeted for detection in this study and no active efflux contribution of PAβN-inhibited efflux pumps to carbapenem resistance was detected, other potential chromosomal mutations such as reduced expression or alteration in outer membrane porin or penicillin-binding protein may have occurred in NC-CRKP to develop carbapenem resistance, as only 46.3% (25/54) of them had porin loss. The majority (53.7%) of the isolated strains in this study did not have porin loss, unlike a multicenter study in Taiwan where only 5.1% (5/99 isolates) of their NC-CRKP isolates preserved expression of both ompK35 and ompK36 porins [18]. The majority of their NC-CRKP conferred carbapenem resistance through a combination of porin loss and acquired non-carbapenemase resistance mechanisms [18].

For PFGE dendrogram analysis, 46 pulsotypes were detected among 53 NC-CRKP. They were grouped into six clusters based on an 80.0% similarity. Cluster D was the dominant strain cluster found in 24 strains (pulsotypes KP 18 to KP 34) isolated from 2013 to 2019. No clonal transmission was identified as all the profiles were unique. This was consistent with the recommendations for the control of carbapenemase-producing *Enterobacteriaceae* published by the Australian Commission on Safety and Quality in Health Care, in which NC-CRKP-infected/colonised patients represented a lower infection control risk and did not warrant attention unless cross-transmission is demonstrated [20]. There was only sporadic isolation of pulsotypes KP24 (six strains), KP26 (two strains), and KP32 (two strains). No circulation of a specific pulsotype occurred in our study setting. Nonetheless, the high diversity observed may be indicative of the presence of the genetic events that contributed to the clone emergence and adaptation to the hospital’s environment.

The possible risk factors previously reported for in-hospital mortality among CRKP-infected/colonised patients were the usage of mechanical ventilation [8] and delayed definitive treatment [9]. Additionally, patients with NDM-producing CRKP had previously been found to have a lower hazard ratio for in-hospital mortality as compared to the OXA-48 variant [10]. In this study, no statistically significant risk factor was found in the multivariate binary logistic regression analysis due to the limited sample size. Therefore, all possibilities remained. A larger sample size should be included in future studies to increase the robustness of the statistical analyses. Additionally, the role of structural mutation such as reduced expression or alteration of porin or penicillin-binding protein should be investigated further to assess the mechanism of carbapenem resistance of the remaining strains (53.7%). Since only the five clinically important major carbapenemase genes (*bla*_NDM_, *bla*_OXA-48_, *bla*_IMP_, *bla*_VIM_, and *bla*_KPC_) were targeted for detection in this study, future studies may also target for detection of rare carbapenemase genes (such as *bla*_IMI_, *bla*_NMC_, *bla*_FRI_, *bla*_GES_, *bla*_BKC_, *bla*_SFC_, *bla*_SME_, *bla*_GIM_, *bla*_TMB_, *bla*_LMB_, *bla*_KHM_, *bla*_SFH_, *bla*_AIM_, *bla*_OXA-372_, *bla*_CMY_, *bla*_ACT_, or *bla*_BIC_) [43] among the isolates in this study.

## 4. Materials and Methods

### 4.1. Bacterial Strains Collection

CRKP was defined as *K. pneumoniae* with a MIC of ≥2 µg/mL for ertapenem or ≥4 µg/mL for meropenem, imipenem, or doripenem [19]. NC-CRKP was classified based on the absence of the five major carbapenemase genes, which were *bla*_NDM_, *bla*_OXA-48_, *bla*_IMP_, *bla*_VIM_, and *bla*_KPC_. The NC-CRKP (clinical and screening) isolated from the University of Malaya Medical Centre (UMMC) patients by the hospital’s Medical Microbiology Diagnostic Laboratory (MMDL) from January 2013 to October 2019 were collected and revived from stock cultures. Only the first isolate per patient that was stocked was included in this collection. The strain identification was performed using the Vitek^®^ 2 system (bioMérieux, Inc., Durham, NC, USA) or the Vitek^®^ MS (bioMérieux, USA). The purity of the strains was checked before the commencement of genotypic analysis.

### 4.2. Determination of Minimum Inhibitory Concentration (MIC) Profiles

The MIC for CIP, IPM, MEM, ETP, and CT were determined using the broth microdilution method in accordance with CLSI guidelines [19]. *Pseudomonas aeruginosa* ATCC27853, *Escherichia coli* ATCC25922 and *Enterococcus faecalis* ATCC29212 were used as quality control strains.

### 4.3. Antimicrobial Susceptibility Data

The antimicrobial susceptibility data of the isolates to seven different antimicrobials (amoxicillin/clavulanate, ampicillin, cefuroxime, ceftazidime, cefotaxime, ceftriaxone, and gentamicin) previously performed by the MMDL, UMMC using the automated Vitek^®^ 2 system (±E-test when required) were retrieved from the MMDL’s laboratory information system.

### 4.4. Determination of Resistance Genes and Porin-Associated Genes

The presence of carbapenemase genes, which were *bla*_KPC_ [44], *bla*_OXA-48_ [45], *bla*_IMP_ [46], *bla*_VIM_ [47], and *bla*_NDM_ [47]; the *AmpC* β-lactamase genes, which were *bla*_DHA_, *bla*_CMY_, *bla*_FOX_, and *bla*_ACT_ [48]; the other non-carbapenemase β-lactamase genes, which were *bla*_TEM_ [48], *bla*_SHV_ [48], *bla*_CTX-M_ (CTX-M-1 group) [49], *bla*_OXA-1_ [50], and *bla*_OXA-9_ [45]; as well as the porin-associated genes, which were *ompK35*, *ompK36*, and *ompK37* [51], were determined via polymerase chain reaction (PCR).

### 4.5. MIC Reduction Assay with Efflux Pump Inhibitor

For efflux pump contribution, the MICs of IPM, MEM, and ETP in combination with 26.3 µg/mL phenylalanine-arginine β-naphthylamide (PAβN; TargetMol, Wellesley Hills, MA, USA), an efflux pump inhibitor, were determined [40]. The MIC for CIP in combination with PAβN was also quantified and used as a control since efflux pumps are involved in the quinolone resistance in *K. pneumoniae*. A ≥4-fold decrease in MIC after the addition of PAβN was considered significant [40,52].

### 4.6. Pulsed-Field Gel Electrophoresis (PFGE)

The genetic relatedness of the NC-CRKP was evaluated using pulsed-field gel electrophoresis (PFGE). PFGE was performed in accordance with the protocol from PulseNet [53]. Entire bacterial genomic DNA was resolved in 1.0% of Type 1 agarose in CHEF Mapper^®^ XA System (Bio-Rad, Hercules, CA, USA). The PFGE banding patterns were analysed by using BioNumerics version 7.6 software (Applied Maths NV, Sint-Martens-Latem, Belgium) [54]. The band matching was performed at 1.0% trace-to-trace optimisation with a position tolerance of 1.5%.

### 4.7. Clinical Data and Statistical Analysis

The patient’s clinical data, including demographic data, underlying diseases, previous hospitalisation within one year, period of hospitalisation before isolation of positive cultures, intubations, previous antimicrobial exposure within 90 days, empirical treatment, definitive therapy, and the infection-acquired model with outcomes of antimicrobial therapies were extracted from the hospital electronic medical record (EMR) database. Data were anonymised to protect confidentiality. The following criteria were defined: (1) Patients were classified as having an infection if there was clinical or biochemical evidence of infection [55], and as colonization, if the isolation of the microorganism was from non-sterile body sites (rectum/perianal area, respiratory aspirate, vagina, skin, and urine) and there was neither signs nor symptoms of infection [56]. (2) All-cause in-hospital mortality was defined as any death that occurred throughout the hospitalisation from admission until discharge, regardless of cause [57]. (3) Previous antimicrobial exposure was defined as the antimicrobial used within the previous three months. (4) Empiric treatment was defined as the initial antimicrobial administered after the onset of bacteremia and before the microbiological results were available [58]. (5) Definitive therapy was defined as the antimicrobial given to the patients after the microbiological susceptibility results were available [59]. (6) Invasive device was defined as a medical device that is introduced into the body through a body orifice or a breach in the skin. This included chest tube, common bile duct (CBD) tube, colostomy bag, rectal tube, Ryles tube, nasogastric tube, central venous line (CVL), peripheral line, arterial line, peripherally inserted central catheter (PICC) line, intravenous (IV) line, internal jugular catheter (IJC), urinary catheter, pigtail catheter, condom catheter, cannula, and branula. (7) Invasive procedure was defined as one where purposeful/deliberate access to the body is gained via an incision, percutaneous puncture, where instrumentation is used in addition to the puncture needle or instrumentation via a natural orifice by trained healthcare professionals [60]. This included stoma, tracheostomy, cystoscopy, laparotomy, bronchoscopy, colostomy, total abdominal hysterectomy bilateral salpingo-oophorectomy (TAHBSO), endoscopic retrograde cholangiopancreatography (ERCP), and percutaneous transhepatic biliary drainage (PTBD). (8) Based on the Charlson Comorbidity Index (CCI) score category [61], the severity of comorbidity was categorised into three grades: mild, with CCI scores of 1–2; moderate, with CCI scores of 3–4; and severe, with CCI scores of ≥ 5 [62,63]. CCI was calculated according to the scoring system established by Charlson et al. (1987).

Statistical analyses were performed with IBM SPSS (Statistical Package for the Social Sciences) statistics software, version 27.0 (IBM, Armonk, NY, USA). Depending on the normality of the data and the level of measurements, the risk factors of NC-CRKP associated with all-cause in-hospital mortality were analysed independently using the X^2^ test (categorical variables), Fisher’s exact test (categorical variables), Mann–Whitney U test (continuous variables) or Student’s *t*-test (continuous variables). A binary logistic regression model was built to evaluate the risk for all-cause in-hospital mortality among NC-CRKP patients. All tests with a *p*-value of less than 0.050 were considered statistically significant. An odds ratio of <1 indicated a lower risk of all-cause in-hospital mortality, while an odds ratio of >1 indicated a higher risk of all-cause in-hospital mortality.

## 5. Conclusions

An increasing trend of NC-CRKP prevalence was observed over the seven years from January 2013 to October 2019. In this study, 23 (44.2%) patients were infected with NC-CRKP, while others were colonised (55.8%, 29/52). The all-cause in-hospital mortality rate was 46.2% (24/52). All NC-CRKP in this study were MDR and carried at least one non-carbapenemase β-lactamase gene targeted for detection in this study. None of the isolated strains conferred carbapenem resistance through the efflux pumps system. In this study, only 25 (46.3%) NC-CRKP conferred carbapenem resistance through a combination of porin loss and the acquisition of non-carbapenemase resistance mechanisms. Different chromosomal mutations combined with the acquisition of non-carbapenemase resistance mechanisms may have been utilised by the remaining strains (53.7%). Other potential chromosomal mutation mechanisms, such as reduced expression or alteration in outer membrane porin or penicillin-binding protein that may contribute to carbapenem resistance, should be further investigated. Furthermore, the presence of rare carbapenemase genes, which were not targeted for detection in this study, also might be one of the possible resistance mechanisms. Therefore, future studies are warranted to confirm our hypothesis. Fortunately, no circulation of specific pulsotype occurred in our study setting, as there was only sporadic isolation of pulsotypes KP24, KP26, and KP32. The emergence of NC-CRKP in recent years requires continued monitoring as they do not harbour any major carbapenemase genes and their mechanisms of carbapenem resistance remain unclear. Rapid identification and distinction of the NC-CRKP mechanisms would allow for optimal treatment and infection control efforts. Heightened awareness and vigilance for NC-CRKP are obligatory.

## Figures and Tables

**Table 1:** Interpretive MIC categories of all 54 NC-CRKP via broth microdilution method.

Antibiotics	Susceptible (S), *n* (%)	Intermediate (I), *n* (%)	Resistant (R), *n* (%)
	MIC ≤ 0.25 µg/mL	MIC = 0.5 µg/mL	MIC ≥ 1 µg/mL
Ciprofloxacin (CIP)	0 (0.0)	7 (13.0)	47 (87.0)
	MIC ≤ 1 µg/mL	MIC = 2 µg/mL	MIC ≥ 4 µg/mL
Imipenem (IPM)	33 (61.1)	5 (9.3)	16 (29.6)
Meropenem (MEM)	18 (33.3)	9 (16.7)	27 (50.0)
	MIC ≤ 0.5 µg/mL	MIC = 1 µg/mL	MIC ≥ 2 µg/mL
Ertapenem (ETP)	0 (0.0)	2 (3.7)	52 (96.3)
	-	MIC ≤ 2 µg/mL	MIC ≥ 4 µg/mL
Colistin (CT)	-	52 (96.3)	2 (3.7)

The symbol “-” denotes not applicable.

**Table 2:** Frequency of carbapenem resistance among 54 NC-CRKP.

Frequency of Carbapenem Resistance	Number of Strains, *n* (%)		Loss of ompK35 Porin, *n* (%)		Loss of ompK36 Porin, *n* (%)		Loss of Both ompK35 and ompK36 Porins, *n* (%)	
Mono-resistant to IPM	0	(0.0)	-	-	-	-	-	-
Mono-resistant to MEM	0	(0.0)	-	-	-	-	-	-
Mono-resistant to ETP	16	(29.6)	0	(0.0)	6	(11.1)	0	(0.0)
Resistant to IPM, MEM, and ETP	19	(35.2)	2	(3.7)	5	(9.3)	1	(1.9)
Resistant to MEM and ETP only	17	(31.5)	0	(0.0)	10	(18.5)	0	(0.0)
Resistant to IPM and ETP only	2	(3.7)	0	(0.0)	1	(1.9)	0	(0.0)
Total	54	(100.0)	2	(3.7)	22	(40.7)	1	(1.9)

The symbol “-” denotes not applicable.

**Table 3:** Antimicrobial sensitivity of all 54 strains via the automated Vitek^®^2 system.

Antimicrobials		Number of Strains, *n*		
		Susceptible (S)	Intermediate (I)	Resistant (R)
Amoxicillin clavulanate	(AMC)	0	1	53
Ampicillin	(AMP)	0	0	54
Cefuroxime	(CXM)	0	0	54
Ceftazidime	(CAZ)	2	0	52
Cefotaxime	(CTX)	1	1	52
Ceftriaxone	(CRO)	3	1	50
Gentamicin	(GM)	30	0	24

**Table 4:** Detection of resistance genes and porin-associated genes of all 54 strains.

Resistance Genes (Either Alone or Co-Carried)	Porin Loss (ompK)	Number of Strains, *n* (%)	Resistance Profile *
*bla*_KPC_	-	0 (0.0)	-
*bla*_OXA-48_	-	0 (0.0)	-
*bla*_VIM_	-	0 (0.0)	-
*bla*_IMP_	-	0 (0.0)	-
*bla*_NDM_	-	0 (0.0)	-
*bla*_CMY_	-	0 (0.0)	-
*bla*_FOX_	-	0 (0.0)	-
*bla*_ACT_	-	0 (0.0)	-
*bla*_SHV_	-	1 (1.9)	CAZ, CTX, CRO, GM
*bla*_SHV_	36	1 (1.9)	CAZ, IPM
*bla*_SHV_ and *bla*_CTX-M_	-	11 (20.4)	CAZ, CTX, CRO
*bla*_SHV_ and *bla*_CTX-M_	36	11 (20.4)	CAZ, CTX, CRO
*bla*_CTX-M_ and *bla*_TEM_	-	1 (1.9)	CAZ, CTX, CRO, MEM
*bla*_SHV_ and *bla*_TEM_	-	1 (1.9)	CAZ, CTX, CRO, MEM, IPM, GM
*bla*_SHV_ and *bla*_TEM_	36	1 (1.9)	CTX, CRO
*bla*_SHV_, *bla*_CTX-M_, and *bla*_TEM_	-	7 (13.0)	CAZ, CTX, MEM
*bla*_SHV_, *bla*_CTX-M_, and *bla*_TEM_	35	1 (1.9)	CAZ, CTX, CRO, MEM, IPM
*bla*_SHV_, *bla*_CTX-M_, and *bla*_TEM_	36	6 (11.1)	CAZ, CTX, CRO, MEM
*bla*_SHV_ and *bla*_OXA-1_	36	1 (1.9)	CTX, GM
*bla*_SHV_, *bla*_CTX-M_, *bla*_TEM_, and *bla*_OXA-1_	35	1 (1.9)	CAZ, CTX, CRO, MEM, IPM,
*bla*_SHV_, *bla*_CTX-M_, *bla*_OXA-1_, and *bla*_OXA-9_	-	1 (1.9)	CAZ, CTX, CRO, MEM, GM
*bla*_SHV_, *bla*_CTX-M_, *bla*_TEM_, and *bla*_OXA-9_	-	2 (3.7)	CAZ, CTX, CRO, MEM
*bla*_SHV_ and *bla*_DHA_	-	2 (3.7)	CAZ, CTX, CRO, MEM, IPM
*bla*_SHV_ and *bla*_DHA_	36	1 (1.9)	CAZ, CTX, CRO
*bla*_SHV_, *bla*_CTX-M_, and *bla*_DHA_	-	1 (1.9)	CAZ, CTX, CRO, IPM, GM
*bla*_SHV_, *bla*_CTX-M_, *bla*_TEM_, and *bla*_DHA_	-	2 (3.7)	CAZ, CTX, CRO
*bla*_SHV_, *bla*_CTX-M_, *bla*_TEM_, and *bla*_DHA_	36	1 (1.9)	CAZ, CTX, CRO
*bla*_SHV_, *bla*_CTX-M_, *bla*_TEM_, and *bla*_DHA_	35, 36	1 (1.9)	CAZ, CTX, CRO, MEM, IPM

* All strains were resistant to ETP, CIP, AMC, AMP, and CXM. Presence of resistance genes (either alone or co-carried), such as the carbapenemase genes: *bla*_KPC_, *bla*_OXA-48_, *bla*_IMP_, *bla*_VIM_, and *bla*_NDM_; the *AmpC* β-lactamase genes: *bla*_DHA_, *bla*_CMY_, *bla*_FOX_, and *bla*_ACT_; and the other non-carbapenemase β-lactamase genes: *bla*_TEM_, *bla*_SHV_, *bla*_CTX-M_, *bla*_OXA-1_, and *bla*_OXA-9_. Resistance profile: Ertapenem (ETP), imipenem (IPM), meropenem (MEM), colistin (CT), ciprofloxacin (CIP), amoxicillin clavulanate (AMC), ampicillin (AMP), cefuroxime (CXM), ceftazidime (CAZ), cefotaxime (CTX), ceftriaxone (CRO), or gentamicin (GM). The symbol “-” denotes not applicable.

**Table 5:** Summary of MIC of carbapenem and ciprofloxacin in the presence of PAβN among all 54 NC-CRKP.

Characteristics	No. of Strains			
	CIP	IPM	MEM	ETP
Susceptible strain	0	33	18	0
Strains with unchanged MIC	30	10	16	12
Strains with a ≥2-fold decrease in MIC	24	1	-	-
Strains with a ≥2-fold increase in MIC	-	10	20	42
Total	54	54	54	54
Strains with a ≥4-fold decrease in MIC	5 *	-	-	-
Strains with a ≥4-fold increase in MIC	-	4	7	20

* A four-fold decrease in MIC after the addition of PAβN was considered significant. The symbol “-” denotes not applicable.

**Table 6:** Risk factors of NC-CRKP associated with all-cause in-hospital mortality by univariate analysis.

Variable	Case		All-Cause In-Hospital Mortality				*p*-Value
			Yes		No		
	*n*	(%)	*n*	(%)	*n*	(%)	
NC-CRKP model, Case, *n* = 52 (%)							
Age (years old), mean (SD)	57.4	(20.3)	61.4	(16.1)	53.9	(23.1)	0.189 ^c^
Gender:							
Male	23	(44.2)	8	(34.8)	15	(65.2)	0.143 ^a^
Female	29	(55.8)	16	(55.2)	13	(44.8)	
Ethnic:							0.476 ^a^
Malay	17	(32.7)	8	(47.1)	9	(52.9)	
Chinese	19	(36.5)	11	(57.9)	8	(42.1)	
Indian	13	(25.0)	4	(30.8)	9	(69.2)	
Others	3	(5.8)	1	(33.3)	2	(66.7)	
Model:							
Infection	23	(44.2)	12	(52.2)	11	(47.8)	0.438 ^a^
Colonisation	29 ^e^	(55.8)	12	(41.4)	17	(58.6)	
Length of hospitalization (days), mean (SD)	40.5	(32.0)	36.0	(29.6)	44.3	(33.9)	0.388 ^d^
ICU stay before strain isolation	32	(61.5)	16	(50.0)	16	(50.0)	0.482 ^a^
Blood transfusion	35	(67.3)	19	(54.3)	16	(45.7)	0.091 ^a^
Mechanical ventilation	43	(82.7)	22	(51.2)	21	(48.8)	0.152 ^b^
Invasive procedures	45	(86.5)	23	(51.1)	22	(48.9)	0.107 ^b^
Invasive devices	52	(100.0)	24	(46.2)	28	(53.8)	N.A.
Comorbidities:							
Autoimmune disease	2	(3.8)	0	(0.0)	2	(100.0)	0.493 ^b^
Malignancy	21	(40.4)	12	(57.1)	9	(42.9)	0.191 ^a^
Cardiovascular disease	15	(28.8)	8	(53.3)	7	(46.7)	0.508 ^a^
Cerebrovascular accident	8	(15.4)	3	(37.5)	5	(62.5)	0.711 ^b^
Diabetes mellitus	25	(48.1)	13	(52.0)	12	(48.0)	0.416 ^a^
Hypertension	29	(55.8)	14	(48.3)	15	(51.7)	0.730 ^a^
Renal disease (CKD/ESRF)	13	(25.0)	8	(61.5)	5	(38.5)	0.199 ^a^
Mental and behavioural disorders	5	(9.6)	4	(80.0)	1	(20.0)	0.169 ^b^
Hematological disorders	9	(17.3)	4	(44.4)	5	(55.6)	1.000 ^b^
Liver disease	6	(11.5)	2	(33.3)	4	(66.7)	0.674 ^b^
Peptic ulcer disease	4	(7.7)	2	(50.0)	2	(50.0)	1.000 ^b^
Admission diagnosed:							
Respiratory: Pneumonia	15	(28.8)	6	(40.0)	9	(60.0)	0.571 ^a^
Respiratory: Chronic obstructive pulmonary disease (COPD)	2	(3.8)	1	(50.0)	1	(50.0)	1.000 ^b^
Urogenital-related infection	7	(13.5)	4	(57.1)	3	(42.9)	0.690 ^b^
Neurological disease	5	(9.6)	2	(40.0)	3	(60.0)	1.000 ^b^
Trauma	5	(9.6)	3	(60.0)	2	(40.0)	0.652 ^b^
Severity of comorbidity based on Charlson Comorbidity Index (CCI):							
Severe (CCI scores ≥ 5)	32	(61.5)	17	(53.1)	15	(46.9)	0.202 ^a^
Moderate (CCI scores 3–4)	8	(15.4)	6	(75.0)	2	(25.0)	0.123 ^b^
Mild (CCI scores 1–2)	8	(15.4)	1	(12.5)	7	(87.5)	0.056 ^b^
No comorbidity (CCI scores 0)	4	(7.7)	0	(0.0)	4	(100.0)	0.115 ^b^
Resistance genes:							
*bla*_TEM_	23	(44.2)	13	(56.5)	10	(43.5)	0.182 ^a^
*bla*_SHV_	51	(98.1)	24	(47.1)	27	(52.9)	1.000 ^b^
*bla*_CTX-M_	44	(84.6)	19	(43.2)	25	(56.8)	0.447 ^b^
*bla*_OXA-1_	3	(5.8)	2	(66.7)	1	(33.3)	0.590 ^b^
*bla*_OXA-9_	2	(3.8)	1	(50.0)	1	(50.0)	1.000 ^b^
*bla*_DHA_	8	(15.4)	2	(25.0)	6	(75.0)	0.262 ^b^
Loss of *ompK35*	3	(5.8)	3	(100.0)	0	(0.0)	0.092 ^b^
Loss of *ompK36*	23	(44.2)	8	(34.8)	15	(65.2)	0.143 ^a^
Loss of *ompK37*	0	(0.0)	0	(0.0)	0	(0.0)	N.A.
Resistance profile:							
R to CIP	52	(100.0)	24	(46.2)	28	(53.8)	N.A.
R to IPM	20	(38.5)	12	(60.0)	8	(40.0)	0.113 ^a^
R to MEM	34	(65.4)	18	(52.9)	16	(47.1)	0.177 ^a^
R to ETP	52	(100.0)	24	(46.2)	28	(53.8)	N.A.
R to CT	2	(3.8)	1	(50.0)	1	(50.0)	1.000 ^b^
Mono-resistant to ETP	16	(30.8)	5	(31.3)	11	(68.7)	0.151 ^a^
Resistant to MEM and ETP only	16	(30.8)	7	(43.8)	9	(56.2)	0.817 ^a^
Resistant to IPM and ETP only	2	(3.8)	1	(50.0)	1	(50.0)	1.000 ^b^
Resistant to IPM, MEM, and ETP	18	(34.6)	11	(61.1)	7	(38.9)	0.115 ^a^
Previous antimicrobial exposure ^f^:							
Penicillin	9	(17.3)	5	(55.6)	4	(44.4)	0.716 ^b^
β-lactamase inhibitors	39	(75.0)	18	(46.2)	21	(53.8)	1.000 ^a^
Cephems/Cephalosporins	36 ^g^	(69.2)	12	(33.3)	24	(66.7)	0.005 ^a^*
Carbapenem	27 ^h^	(51.9)	17	(63.0)	10	(37.0)	0.012 ^a^*
Fluoroquinolones	7	(13.5)	1	(14.3)	6	(85.7)	0.107 ^b^
Aminoglycosides	7	(13.5)	2	(28.6)	5	(71.4)	0.430 ^b^
Macrolides	5	(9.6)	2	(40.0)	3	(60.0)	1.000 ^b^
Glycopeptides	19	(36.5)	12	(63.2)	7	(36.8)	0.062 ^a^
Nitroimidazoles (Flagyl)	24	(46.2)	10	(41.7)	14	(58.3)	0.548 ^a^
Polymyxins	3	(5.8)	0	(0.0)	3	(100.0)	0.240 ^b^
Folate pathway inhibitors	5	(9.6)	2	(40.0)	3	(60.0)	1.000 ^b^
Infection model, Case, *n* = 23 (%)							
Empiric treatment ^f^:							
β-lactamase inhibitors	6	(26.1)	2	(33.3)	4	(66.7)	0.371 ^b^
Cephems/Cephalosporins	4	(17.4)	2	(50.0)	2	(50.0)	1.000 ^b^
Carbapenem	11	(47.8)	7	(63.6)	4	(36.4)	0.292 ^a^
Aminoglycosides (Amikacin)	1	(4.3)	1	(100.0)	0	(0.0)	1.000 ^b^
Folate pathway inhibitors (Cotrimoxazole)	1	(4.3)	1	(100.0)	0	(0.0)	1.000 ^b^
Nitroimidazoles (Flagyl)	1	(4.3)	1	(100.0)	0	(0.0)	1.000 ^b^
Definitive therapy:							
Carbapenem only	4	(17.4)	1	(25.0)	3	(75.0)	0.317 ^b^
Cotrimoxazole only	1	(4.3)	0	(0.0)	1	(100.0)	0.478 ^b^
Polymyxins and carbapenem	14	(60.9)	8	(57.1)	6	(42.9)	0.680 ^b^
Polymyxins and gentamicin	1	(4.3)	0	(0.0)	1	(100.0)	0.478 ^b^
Nil ^i^	3	(13.0)	3	(100.0)	0	(0.0)	0.217 ^b^
Type of infection:							
Bacteremia	10	(43.5)	6	(60.0)	4	(40.0)	0.680 ^b^
Urinary tract infection	2	(8.7)	0	(0.0)	2	(100.0)	0.217 ^b^
Intra-abdominal sepsis	6	(26.1)	4	(66.7)	2	(33.3)	0.640 ^b^
Skin and soft tissue infection	4	(17.4)	2	(50.0)	2	(50.0)	1.000 ^b^
Pneumonia	1	(4.3)	0	(0.0)	1	(100.0)	0.478 ^b^
Severity of comorbidity based on Charlson Comorbidity Index (CCI):							
Severe (CCI scores ≥ 5)	13	(56.5)	8	(61.5)	5	(38.5)	0.414 ^b^
Moderate (CCI scores 3–4)	4	(17.4)	3	(75.0)	1	(25.0)	0.590 ^b^
Mild (CCI scores 1–2)	5	(21.7)	1	(20.0)	4	(80.0)	0.155 ^b^
No comorbidity (CCI scores 0)	1	(4.3)	0	(0.0)	1	(100.0)	0.478 ^b^

* *p* < 0.050. ^a^ *p*-value obtained using chi-square test. ^b^ *p*-value obtained using Fisher’s exact test. ^c^ *p*-value obtained using Student’s *t*-test. ^d^ *p*-value obtained using Mann–Whitney U Test. ^e^ Among 29 NC-CRKP-colonised patients, 23 were screening samples (rectal) while 6 were clinical samples (4 strains isolated from wound swabs and 2 strains isolated from urine). ^f^ The antimicrobial agent given was either alone or in combination. ^g^ Among 36 NC-CRKP-infected/colonised patients with previous cephems/cephalosporins exposure, 21.1% (4/19) of the NC-CRKP-colonised patients died, while 47.1% (8/17) of the NC-CRKP-infected patients died. They were previously exposed to second- (cefuroxime:12), third- (cefoperazone:20, cefotaxime:1, ceftazidime:3, and ceftriaxone:10), and fourth-generation cephalosporins (cefepime:6). ^h^ Among 27 NC-CRKP-infected/colonised patients with previous carbapenem exposure, 57.9% (11/19) of the NC-CRKP-colonised patients died, while 75.0% (6/8) of the NC-CRKP-infected patients died. They were previously exposed to meropenem (23), imipenem (5), and ertapenem (2). ^i^ Definitive therapy was not administered as the patient underwent amputation or the patient passed away before definitive therapy was initiated. “N.A.” is an abbreviation for not applicable.

**Table 7:** Binomial logistic regression model for all-cause in-hospital mortality among all NC-CRKP patients.

Variable of Binomial Logistic Regression with *p* < 0.150, Case, *n* = 52	*p*-Value	Odds Ratio	95.0% Confidence Interval		
Gender: Male	0.762	0.77	0.14	–	4.20
Blood transfusion	0.356	2.69	0.33	–	22.06
Invasive procedures	0.980	0.97	0.07	–	14.05
Severity of comorbidity based on Charlson Comorbidity Index (CCI):					
Moderate (CCI scores 3–4)	0.216	5.37	0.38	–	76.85
Mild (CCI scores 1–2)	0.254	0.18	0.01	–	3.38
No comorbidity (CCI scores 0)	0.999	0.00	0.00		
Loss of *ompK35*	0.999	788,381,237.10	0.00		
Loss of *ompK36*	0.141	0.24	0.04	–	1.60
Resistance profile:					
R to IPM	0.699	5.48	0.00	–	30,208.78
Resistant to IPM, MEM, and ETP	0.747	0.24	0.00	–	1311.89
Previous antimicrobial exposure:					
Cephems/Cephalosporins	0.124	0.18	0.02	–	1.60
Carbapenem	0.207	3.30	0.52	–	21.04
Fluoroquinolones	0.136	0.12	0.01	–	1.97
Glycopeptides	0.727	1.55	0.13	–	17.89

## Data Availability

Not applicable.

## Appendix A

**Table A1:** MIC of carbapenem and ciprofloxacin in the absence and presence of the efflux pump inhibitor phenylalanine-arginine β-naphthylamide (PAβN) of all 54 NC-CRKP.

Strain No.	MIC (µg/mL)							
	CIP	CIP + PAβN	IPM	IPM + PAβN	MEM	MEM + PAβN	ETP	ETP + PAβN
K1310-23	128	128	2	8	8	16	64	128
B10	128	64 ^a^	0.5	-	2	4	8	32
100	4	1 ^a^*	32	128	64	64	64	256
173	64	32 ^a^	1	-	0.5	-	2	16
174	1	1	4	8	2	2	16	64
175	8	8	2	2	0.5	-	4	16
178	16	8 ^a^	0.5	-	0.5	-	1	4
183	64	64	1	-	0.5	-	16	32
192 ^b^	2	1 ^a^	128	128	64	128	256	256
207	1	0.5 ^a^	1	-	0.5	-	16	64
264	64	32 ^a^	0.5	-	0.125	-	8	8
281	128	128	4	4	8	8	64	64
285	0.5	0.5	2	4	8	8	32	64
1708-4	128	64 ^a^	2	4	16	16	64	128
1708-6	128	64 ^a^	0.25	-	0.25	-	4	8
1708-14	128	64 ^a^	0.25	-	0.125	-	2	4
1708-15	128	64 ^a^	0.5	-	4	4	16	32
1708-27	128	64 ^a^	0.5	-	4	4	32	32
1709-2	128	128	1	-	1	-	8	16
1709-4	64	64	0.5	-	4	4	32	64
1711-2	128	128	4	8	4	16	16	64
1711-4	64	64	0.5	-	2	16	128	128
1711-5 ^b^	64	64	128	128	128	128	256	256
1711-7	64	64	0.5	-	0.25	-	1	8
1712-11	128	128	0.5	-	4	4	16	32
1801-1	128	128	64	128	32	128	128	>256
1801-2	>512	256 ^a*^	0.5	-	0.125	-	16	128
1801-7	128	128	1	-	16	32	64	64
1801-8	64	32 ^a^	0.5	-	2	8	16	64
1802-4	128	128	0.25	-	4	4	32	64
1802-8	128	64 ^a^	32	64	32	64	128	256
1804-1	8	2 ^a*^	1	-	0.5	-	8	32
1805-6	128	64 ^a^	1	-	4	8	32	64
1805-8	2	2	1	-	4	8	16	64
1805-11	32	8 ^a*^	4	4	16	16	128	256
1805-12	8	2 ^a*^	32	32	8	32	128	128
1806-1	128	128	0.5	-	4	4	32	64
1806-4	1	1	0.5	-	2	4	32	32
1808-5	0.5	0.5	1	-	8	16	32	128
1811-5	128	128	1	-	0.25	-	8	8
1811-11	128	64 ^a^	128	128	256	>256	>256	>256
1811-12	8	4 ^a^	8	8	4	16	64	64
1901-7	0.5	0.5	1	-	2	4	4	8
1901-9	4	4	2	2	2	4	16	32
1901-14	128	128	0.25	-	4	4	32	64
1902-6	128	64 ^a^	1	-	0.25	-	4	16
1903-5	0.5	0.5	4	4	8	8	32	128
1904-1	0.5	0.5	1	-	0.25	-	8	16
1905-2	128	64 ^a^	1	-	0.125	-	2	4
1908-3	0.5	0.5	4	16	8	32	128	256
1909-6	2	1 ^a^	1	-	1	-	4	16
1909-8	2	2	8	4 ^a^	2	4	2	32
1909-9	2	2	4	32	0.125	-	8	>256
1910-4	0.5	0.5	0.25	-	2	2	16	32

* Strains with a ≥ 4-fold decrease in MIC in the presence of PAβN (26.3 µg/mL). ^a^ Strains with a ≥ 2-fold decrease in MIC in the presence of PAβN (26.3 µg/mL). ^b^ Strains that were resistant to colistin with a MIC of 32 µg/mL. The symbol “-” denotes not applicable.
